# Stress and PTSD levels among Hamad Medical Corporation Paramedics before and during the COVID-19 pandemic

**DOI:** 10.5339/qmj.2025.111

**Published:** 2025-12-11

**Authors:** Mohammed Jameel Al Barbari, Padarath Gangaram, Hmoud Alolimat, Gary Kenward, James Laughton, Guillaume Alinier

**Affiliations:** 1 Department of Social Work, Doha Institute for Graduate Studies, Doha, Qatar; 2 Hamad Medical Corporation Ambulance Service, Doha, Qatar; 3 Faculty of Health Sciences, Durban University of Technology, Durban, South Africa; 4 Weill Cornell Medicine-Qatar, Doha, Qatar; 5 School of Health, Medicine, and Life Sciences, University of Hertfordshire, Hatfield, Hertfordshire, UK; 6 Faculty of Health and Life Sciences, Northumbria University, Newcastle, UK *Email: galinier@hamad.qa

**Keywords:** Prehospital, paramedics, work-related stress, PTSD, COVID-19, pandemic

## Abstract

**Background:**

During the Coronavirus disease (COVID-19) pandemic, healthcare workers globally experienced unprecedented levels of stress. However, limited research has focused specifically on paramedics. As frontline workers, paramedics were placed under immense and unprecedented pressure. This study assessed stress levels and Post-Traumatic Stress Disorder (PTSD) symptoms among paramedics in Qatar before and during the COVID-19 pandemic.

**Methods:**

An online survey adapted from validated tools was distributed to all paramedics in Qatar employed by Hamad Medical Corporation Ambulance Service (*n* = 1100) between December 2020 and March 2021. Responses from volunteer participants were analyzed with data stratified by demographic factors to explore their impact on self-reported stress and PTSD levels. Stress levels were categorized into five ranges, from low to potentially dangerous.

**Results:**

The study received 272 valid responses. Before the pandemic, paramedics reported moderate stress levels, which escalated to severe levels during the pandemic. A paired t-test revealed significant differences in average stress and PTSD symptom scores before and during the pandemic. Symptoms included intrusive memories, avoidance behaviors, hypervigilance, sleep disturbances, and emotional numbness (*P* < 0.05). The average total PTSD score increased significantly during the pandemic from 37.59 (±16.02) before the pandemic to 42.42 (± 17.29; *P* < 0.05). The proportion of paramedics reporting little to no PTSD symptoms decreased from 39.3% before to 28.3% during the pandemic. Conversely, those reporting highly severe PTSD increased from 33.1% to 44.1% (*P* < 0.001).

**Conclusion:**

The study highlights a significant increase in stress levels and PTSD symptoms among paramedics in Qatar during the COVID-19 pandemic compared to the pre-pandemic period. This rise can be attributed to their role as frontline responders, which exposed them to heightened risks of infection, long shifts, and prolonged periods between leave. The findings underscore the need for targeted interventions to address stress and PTSD among paramedics, ensuring their mental and physical well-being and enabling them to continue providing effective medical care.

## 1. INTRODUCTION

Paramedics, as frontline responders to emergencies, are routinely exposed to high levels of stress, placing them at significant risk for mental health issues such as depression, opioid abuse, and even suicide.^1^ The COVID-19 pandemic emerged from Wuhan, China, in December 2019, exacerbating these challenges, causing widespread fear, panic, and millions of deaths globally.^2^ Healthcare professionals, particularly those directly involved in pandemic response efforts, faced unprecedented levels of anxiety and stress. This was driven by the unknown nature of the virus, the rapidly increasing number of infections, overwhelmed healthcare systems, and the associated increase in patient workload.^3–5^ Additionally, healthcare workers faced increased mental strain due to the fear of transmitting the virus to their families, particularly in the early stages of the pandemic when personal protective equipment (PPE) was scarce, and vaccines were not yet available.^6–9^

The constant exposure to increased levels of work-related stress during the pandemic led to significant psychological, emotional, and physical challenges for healthcare workers.^10^ The invisible threat of the virus, combined with the risk of infection through direct or indirect patient contact, further compounded these difficulties.^3^ Tragically, many healthcare practitioners worldwide lost their lives while working through the pandemic.^11,12^ For instance, in Italy, early reports indicated that approximately 20% of healthcare workers were infected, with some succumbing to the virus.^13^

The COVID-19 pandemic has significantly impacted healthcare systems worldwide, placing immense psychological strain on frontline healthcare workers. Nurses have faced heightened levels of stress, burnout, and emotional exhaustion due to the unpredictable nature of the virus, increased workload, and fear of infection.^14^ A study conducted in Wuhan and Shanghai during the initial stages of the pandemic revealed that nurses experienced considerable psychological distress, with many adopting various coping strategies to manage their mental health.^14^ This finding aligns with global research highlighting the critical importance of addressing mental health challenges among healthcare professionals during public health crises. In Qatar, where the first COVID-19 cases were reported in March 2020, similar psychological burdens were observed among frontline nurses.^15^ Despite their crucial role in prehospital care, limited research has specifically explored the impact of the pandemic on paramedics, particularly in the Middle East.

The Middle East is a region with a high proportion of migrant workers across various sectors, including healthcare.^16^ Many of these migrant workers have families residing in their countries of origin, which may add to their stress during a pandemic. For example, a study by Nashwan et al.^17^ highlighted that nurses in Qatar experienced significant stress, with some considering resignation due to the pandemic’s impact on their mental health. This context underscores the need to explore how paramedics employed by Qatar’s national Ambulance Service, and who are mostly recruited from overseas, perceived the pandemic’s impact on their stress levels and symptoms of Post-Traumatic Stress Disorder (PTSD).

Qatar’s healthcare system is well-developed, supported by advanced technology and processes, including its pre-hospital care services.^18^ The Hamad Medical Corporation Ambulance Service (HMCAS) is a key component of this system, staffed by highly qualified paramedics and equipped with a modern fleet of emergency response vehicles.^19,20^ Paramedics in Qatar must meet stringent qualification requirements and undergo a rigorous onboarding and training program.^21,22^ Additionally, paramedics are required to participate in continuing professional development programs, some of which were specifically tailored to the pandemic, such as training on the proper use of PPE during patient care and transportation.^23^

The pre-hospital environment in which paramedics practice is unique and stressful by nature. Travelling at high speeds to reach the sick and injured in the shortest possible time, the inclement weather, treating critical patients, etc., all contribute to the paramedic’s stressors on a daily basis. Despite having robust systems, paramedics had to now deal with the added stressors of the pandemic while being away from their home country, which likely impacted their mental health. This study aimed to assess the self-perceived levels of stress and PTSD symptoms experienced by paramedics in Qatar before and during the COVID-19 pandemic, providing valuable insights into the mental health challenges faced by this critical workforce.

## 2. METHODS

### 2.1 Study design

This study adopted a descriptive and analytical approach using quantitative methods. An anonymous online survey, hosted on Google Docs, was distributed via a hyperlink within a text message to all staff registered on the HMCAS messaging system (*N* = 1100). The survey assessed socio-demographic factors (e.g., age, gender, years of experience, marital status, and nationality), work-related stress levels before and during the COVID-19 pandemic, and factors associated with PTSD. As the pandemic was not anticipated, there was no other way of determining the participants’ baseline level of stress and anxiety, hence the reliance on retrospective self-reported assessment. Data collection occurred between December 1, 2020, and March 31, 2021. The analysis focused on evaluating the impact of socio-demographic and work-related factors on stress levels and PTSD symptoms, providing insights into how different groups within the study population were affected by the pandemic.

### 2.2 Data collection tool

Some sections of the survey were adapted from validated tools to address the research objectives in the context of the pandemic and the diverse backgrounds of HMCAS pre-hospital healthcare professionals. They included the Workplace Stress Scale: Attitudes in the American Workplace VII^24^ used to measure work-related stress levels, and the PTSD Checklist for DSM-IV (PCL-M),^25^ which is a 17-item survey assessing the severity of PTSD symptoms based on DSM-IV criteria. The data collection tool also included additional demographic questions to capture socio-demographic and work-related information. The first page of the survey provided a detailed study briefing, consent information, and had a stated completion time estimated to be 15 to 20 minutes.

### 2.3 Sampling approach and sample size

All ambulance paramedics (APs) and critical care paramedics (CCPs) employed by HMCAS (*N* = 1100) were invited to participate. Invitations were sent via text messages, and notices with QR codes were displayed in ambulance stations across Qatar. A non-probability purposive sampling method was used to recruit participants.

To determine the required sample size, a power analysis was conducted. Based on an estimated effect size of 0.3 (moderate effect), a significance level (α) of 0.05, and a power (1−β) of 0.80, the calculated required sample size was 272 participants. This sample size was deemed sufficient to detect significant differences in stress levels and PTSD symptoms before and during the pandemic.

### 2.4 Inclusion criteria

Participants were required to meet the following inclusion criteria:

1. Employed as APs or CCPs by HMCAS before December 1, 2019, to ensure they had completed their onboarding training and gained pre-pandemic prehospital care experience in Qatar.
2. Working at least two 12-hour shifts per month responding to emergency calls during the study period.

### 2.5 Statistical analysis and validation

The overall survey was reviewed and refined through a Delphi process involving four senior members from the Ambulance Service with research experience, as well as an academic supervisor. This expert review ensured face validity and contextual appropriateness of the combined tools with the introduction of a demographic information section, particularly as no formal pilot testing was conducted. Additionally, internal consistency of the scales was assessed post-hoc using Cronbach’s alpha, which indicated acceptable reliability levels for both the stress and PTSD items.

The data collected from the electronic survey included socio-demographic factors, work-related stress variables, and coping strategies. Data was entered and analyzed using the Statistical Package for Social Sciences (SPSS, version 25). Descriptive statistics, including frequencies, percentages, means, medians, and ranges, were used to summarize categorical and continuous variables. For categorical variables, chi-square analysis was employed as the primary test of significance. Continuous variables were analyzed using single-sample and paired t-tests, depending on the comparison required. Effect sizes were calculated using Eta squared, and 95% confidence intervals (CI) were reported where applicable. A *P*-value of < 0.05 was considered statistically significant.

### 2.6 Ethical approval and consent process

The study was conducted in full compliance with the principles of the Declaration of Helsinki, Good Clinical Practice (GCP), and the laws and regulations of the Ministry of Public Health in Qatar. Ethical approval was obtained from Doha Institute for Graduate Studies Institutional Review Board (DI-IRB) under approval number DI-IRB-2020-S84 and from Hamad Medical Corporation (HMC) and Medical Research Center (MRC) under approval number MRC-01-20-1156. At the beginning of the online survey, participants were provided with an explanation of the study’s purpose, the anonymous nature of their participation, and their right to withdraw at any time without justification. Everyone was informed that completing the survey was considered voluntary consent to participate.

## 3. RESULTS

The survey was completed by 274 HMCAS staff, of whom 2 were excluded for not working as APs or CCPs. The final sample included 272 participants (Table 1). Key demographic characteristics of the study population are summarized as follows: the majority were male (94.9%), aged 31 to 40 years (68.0%), and of Arabian ethnicity (54.8%). Most participants had over 6 years of clinical experience (90%) and held at least an undergraduate degree. Additionally, 88.2% were married, with 90.1% of married personnel reporting that their spouse resided in Qatar. Notably, 75% of the study population had not tested positive for COVID-19 at the time of data collection.

The self-evaluation of the level of stress experienced by Qatar’s paramedics before and during the COVID-19 pandemic is reported in Table 2. The paired t-test showed that there are statistically significant differences in the average score (out of 5) between before and during the current pandemic for six of the nine questions of the stress evaluation tool. There was a slight increase in relation to staff reporting that the conditions at work were unpleasant or sometimes even unsafe. They felt that work was negatively affecting their physical well-being, their emotional well-being, that they had too much work to do and/or too many unreasonable deadlines, that they found it difficult to express opinions or feelings about work conditions to superiors, and that they felt work pressures interfered with family or personal life (*P* < 0.001). In contrast, the results also showed very similar scores before and during the COVID-19 pandemic in relation to having adequate control or input over work duties, receiving appropriate recognition or rewards for good performance at work, and being able to fully utilize skills and talents at work, but this was not statistically significant. When the responses were analyzed globally, it was also determined that there was a statistically significant difference in the participants’ score of 2.06 points out of a maximum of 45 points between before and during the COVID-19 pandemic, indicating a slightly higher reported level of stress among paramedics during the pandemic (Table 2).

The reported level of stress of the study participants was calculated by compiling their responses in accordance with the Marlin Company and the American Institute of Stress tool,^24^ and results are presented in Table 3. The McNemar-Bowker test showed that the increased level of reported stress during the COVID-19 pandemic was noticeable and statistically significant, as pre-pandemic, 45.6% of paramedics reported a severe to potentially dangerous level of stress (scores of 29–45), and this rose to 62% during the pandemic.

Responses to adapted questions of the tool by Weathers et al.^25^ are presented in Table 4 and demonstrate how staff were mentally or physically affected by the pandemic. The paired t-test showed that there are statistically significant differences in average scores between before and during the COVID-19 pandemic among the study population for all the individual questions. Finally, the overall results for that section of the survey showed that the increase by 4.87 mean points over the total score out of 85 between before and during the pandemic was statistically significant.

Cumulative scores related to participants’ responses to questions focusing on stressful work experiences are presented in Table 5. The results indicate an 11-percentage point increase in the proportion of staff experiencing highly severe PTSD symptoms during the COVID-19 pandemic compared to the pre-pandemic period. Specifically, the percentage of participants with highly severe PTSD symptoms rose from 33.1% before the pandemic to 44.1% during the pandemic. This change was statistically significant as determined by the McNemar-Bowker test.

## 4. DISCUSSION

The COVID-19 pandemic has had far-reaching consequences for frontline workers in both personal and professional lives globally. Notably, physical distancing measures to limit the spread of COVID-19 were practiced in all countries, including Qatar, with the associated and expected physical and mental health issues.^26^ Survey findings from an Australian study involving 95 paramedics revealed that the pandemic significantly disrupted their work-life balance, increased stress and anxiety, and limited their ability to rely on usual coping strategies due to lockdowns and physical distancing restrictions.^27^ Similarly, a study conducted in Spain with 280 paramedics reported a significant negative impact on mental health, including increased stress, emotional exhaustion, depersonalization, and reduced personal accomplishment.^28^

Our study provides additional insights into the impact of the COVID-19 pandemic on pre-hospital care professionals, specifically paramedics in Qatar who are expatriate workers. A questionnaire-based study conducted in Qatar during the pandemic revealed that thanks to the training they received, paramedics had good knowledge regarding the risks of transmission of COVID-19 and the proper use of PPE.^23^ We explored their self-perceived stress levels using the validated Workplace Stress Scale: Attitudes in the American Workplace VII^24^ and self-assessed PTSD symptoms using the PTSD Checklist for DSM-IV (PCL-M).^29^ The participant sample was representative of the overall paramedic population at HMCAS in terms of gender, age, ethnicity, professional experience, and scope of practice. However, the findings are context-specific, reflecting the local working environment, the structure of a governmental pre-hospital care provider, and Qatar’s effective pandemic management measures, which resulted in a relatively low mortality rate.^30^

Comparing stress levels before and during the pandemic, we found a statistically significant 9.3% increase in participants’ reported stress levels based on their responses to nine work-related questions (Table 2). The highest increases in mean scores were related to perceptions of unsafe and unpleasant working conditions, negative impacts on physical and emotional well-being, work pressures interfering with family or personal life, and excessive workload. These findings align with global trends, as the pandemic created a surge in workload for healthcare professionals, leading to emotional, mental, and physical exhaustion.^31^

The proportion of staff reporting a potentially dangerous level of stress increased from 18.8% before the pandemic to 29.4% during the pandemic, reflecting the heightened pressures of the work environment (Table 3). This trend is consistent with findings from Virtanen et al.,^32^ who observed varying stress levels among healthcare workers, with doctors being the most affected. However, our study uniquely compares retrospectively self-reported stress levels before and during the pandemic, providing new insights into the specific impact of COVID-19 on paramedics.

Regarding PTSD symptoms, the proportion of staff reporting few or no symptoms decreased from 39.3% before the pandemic to 28.3% during the pandemic, while those with highly severe PTSD symptoms increased from 33.1% to 44.1% (Table 5). This 11-percentage point increase in severe PTSD symptoms was statistically significant, highlighting the escalating mental health challenges faced by paramedics at HMCAS in Qatar during the pandemic.

This study was conducted during the early stages of Qatar’s vaccination campaign, which began in late February 2021.^33^ While vaccine hesitancy was observed among some healthcare workers in Qatar,^34^ vaccination generally provided a sense of security,^34^ potentially mitigating further increases in stress levels.

The findings align with global trends, where healthcare professionals worldwide reported exacerbated stress and PTSD symptoms during the pandemic. Given these findings, it is imperative to implement targeted interventions to mitigate the effects of increased stress and PTSD among paramedics. Based on related published work, strategies could focus on:

- **Enhancing mental health support systems (e.g., counseling, peer support programs):** Some studies indicate that access to psychological support services, such as counseling and peer support programs, can significantly reduce stress and PTSD symptoms among healthcare professionals.^35–37^
- **Providing trauma-informed care training to address PTSD symptoms:** According to some research, trauma-informed training equips healthcare workers with the skills to recognize and respond to signs of trauma, thereby improving resilience and coping mechanisms.^38–40^ This could easily be implemented by adapting existing online or face-to-face courses, as a survey-based study conducted in Qatar reported relatively high levels of satisfaction among paramedics regarding the online training they attended during the pandemic.^41^
- **Improving workplace safety protocols and reducing excessive workloads:** Although challenging to implement during the pandemic due to unprecedented demands and resource constraints, these remain critical strategies for mitigating stress in high-stress healthcare settings. While full implementation during the acute phases of the pandemic may not have been feasible, research indicates that workload management and well-defined safety procedures can significantly reduce burnout and improve mental health outcomes in high-pressure environments.^29,42,43^ Even incremental measures, such as rotating shifts to prevent overwork or ensuring consistent access to PPE, could have partially alleviated stress during the pandemic while laying the groundwork for long-term systemic improvements.
- **Promoting work-life balance through flexible scheduling and stress management resources:** Though challenging to implement during the pandemic due to heightened demands and staffing shortages, it remains a valuable strategy for enhancing job satisfaction and reducing psychological distress. While full implementation of interventions like flexible shift scheduling may not have been feasible during the acute phases of the pandemic, research highlights their effectiveness in alleviating stress and improving mental health outcomes in high-pressure environments.^10,44,45^ Even limited measures, such as providing access to virtual stress management resources or offering brief mental health breaks during shifts, could have partially supported staff well-being during the pandemic while paving the way for more comprehensive post-crisis interventions.

Failure to address these issues risks long-term adverse outcomes, including burnout, chronic mental health conditions, and compromised quality of patient care. Proactive measures are essential to safeguard paramedics’ well-being and ensure their capacity to deliver effective prehospital care during crises.

### 4.1 Limitations

This study has several limitations that should be acknowledged. First, the study relied on a convenience sample of participants and an online survey, which relied partially on retrospective recall of the level the stress and anxiety they experienced before the pandemic, which may have limited the accuracy and depth of participants’ responses regarding their feelings and experiences before and during the pandemic. Second, there was no question in the validated tool asking participants to disclose if they had previously been diagnosed with or exposed to a specific trauma before the pandemic, which could have triggered PTSD; however, given the common nature of the role of the participants and the appropriate sample size, we believe it to be a non-significant limitation. Third, the use of Google Docs. The survey posed a challenge, as it is blocked on the organization’s internal communication network through which many staff access free Wi-Fi. This likely discouraged some staff from participating, potentially affecting the representativeness of the sample. Fourth, the study’s findings are specific to the context of Qatar’s pre-hospital care system with expatriate paramedics. However, the study findings may be generalizable to other countries in the Middle East with similar healthcare systems that also rely significantly on an expatriate workforce. Future studies could address these limitations by using mixed-method approaches, ensuring broader accessibility to the survey platform, extending the recruitment period to achieve a larger and more diverse sample, and exploring the potential variation of responses by nationality.

## 5. CONCLUSION

The reported level of stress among HMCAS paramedics showed a statistically significant increase during the COVID-19 pandemic compared to the pre-pandemic period. The most concerning findings included:

1. A noticeable rise in the proportion of paramedics experiencing potentially dangerous stress levels, escalating from 18.8% pre-pandemic to 29.4% during the pandemic.
2. A significant increase in highly severe PTSD symptoms, with the percentage of affected staff rising from 33.1% to 44.1%.

These increases underscore the profound challenges faced by paramedics as frontline responders, including heightened exposure to viral infections, traumatic work environments, and sustained psychological strain. The findings align with global trends, where healthcare professionals worldwide reported exacerbated stress levels and PTSD symptoms during the pandemic.

It is recommended that systems be put in place to mitigate the effects of increased stress and PTSD among paramedics as preventive measures to preclude potential long-term adverse outcomes, such as chronic stress, burnout, and chronic mental illnesses, which can all negatively impact patient care.

## ETHICAL APPROVAL

Ethical approval was obtained from Doha Institute for Graduate Studies Institutional Review Board (DI-IRB) under approval number DI-IRB-2020-S84 and the Hamad Medical Corporation (HMC) Medical Research Center (MRC) under approval number MRC-01-20-1156.

## AUTHORS’ CONTRIBUTION

MA: Conceptualization, Methodology, Literature review, Data collection tool design, Data collection, Data analysis, Data interpretation, Writing – original draft. PG: Critical revisions, Data collection, Manuscript review and editing, and Final manuscript approval. HA: Literature review, Data validation, Manuscript review and editing, Final manuscript approval. GK: Critical revisions, Manuscript review and editing, Final manuscript approval. JL: Supervision, Data interpretation, Manuscript review and editing, Final manuscript approval. GA: Supervision, Conceptualization, Literature review, Data collection tool design, Data collection, Data interpretation, Article writing, Final manuscript approval.

## CONFLICT OF INTEREST

The authors declare no conflicts of interest.

## Figures and Tables

**Table 1.** Demographic characteristics of the study participants meet the inclusion criteria.

Baseline characteristics	Frequency	Percentage (%)
**1. In what capacity are you currently working in the Ambulance Service of HMC (HMCAS)?**		
Ambulance paramedic scope of practice	255	93.1
Critical care paramedic scope of practice	17	6.2
**2. Gender**		
Female	14	5.1
Male	258	94.9
**3. What is your ethnicity?**		
Arabian (West Asia and North Africa)	149	54.8
Southeast Asian (Vietnam, Philippines, …)	60	22.1
South Asian (India, Sri Lanka, …)	45	16.5
Caucasian	13	4.8
Black/African	5	1.8
**4. What is your nationality?**		
Tunisian	74	27.2
Jordanian	70	25.7
Filipino	63	23.2
Indian	42	15.4
South African	15	5.5
Other	2	0.7
British	1	0.4
Egyptian	1	0.4
Irish	1	0.4
Palestinian	1	0.4
Somalia	1	0.4
**5. What is your age?**		
21–30 years old	19	7.0
31–40 years old	185	68.0
41–50 years old	55	20.2
51–60 years old	13	4.8
**6. For how many years have you worked for HMCAS?**		
1 to 3 years	46	16.9
4 to 6 years	59	21.7
7 to 10 years	106	39.0
More than 10 years	61	22.4
**7. In total, for how many years have you worked as a clinician?**		
5 years or less	26	9.6
6–10 years	109	40.1
11–15 years	90	33.1
**8. What is your highest educational qualification?**		
Diploma or less	12	4.4
Bachelor	206	75.7
Higher diploma	25	9.2
Masters	17	6.3
PhD or equivalent	12	4.4
**9. What is your marital status?**		
Married	240	88.2
Single	30	11.0
Divorced or separated	2	0.7
**10. If you have a spouse, are they in Qatar**		
No	24	9.9
Yes	218	90.1
**11. Did you test positive for COVID-19 during the current pandemic?**		
Yes	68	25.0
No	204	75.0

**Table 2.** Level of stress reported by Qatar’s paramedics before and during the COVID-19 pandemic based on work-related questions, based on the instrument by Marlin Company and the American Institute of Stress.^24^

Work-related questions	Mean ± SD (*n* = 272)		Mean differences	95% Confidence interval of the difference		Paired t-test	*P*-value
	Before COVID-19	During COVID-19		Lower	Upper		
Were your conditions at work unpleasant or sometimes even unsafe?	3.01 ± 1.04	3.47 ± 1.12	0.46	0.36	0.56	9.417	*P* < 0.001*
Did you feel that your work is negatively affecting your physical well-being?	3.20 ± 1.16	3.64 ± 1.11	0.44	0.34	0.54	9.002	*P* < 0.001*
Did you feel that your work is negatively affecting your emotional well-being?	3.15 ± 1.16	3.59 ± 1.15	0.44	0.35	0.53	9.668	*P* < 0.001*
Did you have too much work to do and/or too many unreasonable deadlines?	3.18 ± 1.13	3.56 ± 1.13	0.38	0.29	0.46	8.689	*P* < 0.001*
Did you find it difficult to express your opinions or feelings about your work conditions to your superiors?	3.13 ± 1.29	3.33 ± 1.31	0.20	0.11	0.29	4.515	*P* < 0.001*
Did you feel that work pressures interfere with your family or personal life?	3.34 ± 1.20	3.74 ± 1.18	0.40	0.31	0.50	8.639	*P* < 0.001*
Did you have adequate control or input over your work duties?^®^	3.21 ± 1.17	3.20 ± 1.22	−0.01	−0.10	0.07	−0.330	*P* = 0.741
Did you receive appropriate recognition or rewards for good performance at work?^®^	2.32 ± 1.20	2.36 ± 1.18	0.04	−0.04	0.13	1.007	*P* = 0.315
Were you able to fully utilize your skills and talents at work?^®^	3.62 ± 1.06	3.58 ± 1.09	−0.04	−0.11	0.03	−1.239	*P* = 0.216
Total score out of 45	27.97 ± 6.85	30.03 ± 7.03	2.06	1.46	2.65	6.841	*P* < 0.001*

Note: Scale is Never = 1, Rarely = 2, Sometimes = 3, Often = 4, Always = 5.

*Significant at *P* ≤ 0.05; *P* > 0.05: Not significant; *n*: number; SD: *s*tandard deviation; ^®^: reverse question.

**Table 3.** Categorized level of stress experienced by Qatar’s paramedics in relation to work before and during the COVID-19 pandemic according to the tool by the Marlin Company and the American Institute of Stress.^24^

Level of stress in relation to work based on the total score	Before COVID-19 (*n* = 272)	During COVID-19 (*n* = 272)	McNemar-Bowker test	*P*-value
	n (%)	n (%)		
Low (17 or less)	20 (7.4)	10 (3.7)	80.052	*P* < 0.001*
Fairly low (18–23)	46 (16.9)	31 (11.4)	
Moderate (24–28)	82 (30.1)	62 (22.8)	
Severe (29–34)	73 (26.8)	89 (32.7)	
Potentially dangerous (35–45)	51 (18.8)	80 (29.4)	
**Total**			**272 (100)**			**272 (100)**			

*Significant difference at *P* ≤ 0.05; *P*-value calculated by McNemar-Bowker test.

**Table 4.** Participants’ responses in relation to how stressful work experiences impacted their mental health before and during the COVID-19 pandemic, based on the tool by Weathers et al.^25^

Questions related to the impact of stressful work experiences on respondents’ mental health	Mean ± SD (*n* = 272)		Mean difference	95% Confidence interval of the difference		Paired t-test	*P*-value
	Before COVID-19	During COVID-19		Lower	Upper		
Did you have repeated, disturbing memories, thoughts, or images of a stressful experience from your work at HMCAS?	2.48 ± 1.15	2.78 ± 1.27	0.31	0.22	0.39	7.113	*P* < 0.001*
Did you have repeated, disturbing dreams of a stressful experience from your work at HMCAS?	2.29 ± 1.21	2.55 ± 1.37	0.26	0.18	0.34	6.404	*P* < 0.001*
Did you suddenly act or feel as if a stressful work experience was happening again (as if you were reliving it)?	2.49 ± 1.26	2.78 ± 1.33	0.29	0.21	0.38	6.644	*P* < 0.001*
Did you feel very upset when something reminded you of a stressful work experience from the past?	2.60 ± 1.36	2.82 ± 1.4	0.21	0.13	0.29	5.249	*P* < 0.001*
Did you have a physical reaction (e.g., heart pounding, trouble breathing, or sweating) when something reminded you of a stressful work experience from the past?	2.03 ± 1.22	2.23 ± 1.3	0.20	0.14	0.27	5.994	*P* < 0.001*
Did you avoid thinking or talking about a stressful HMCAS work experience from the past?	2.38 ± 1.25	2.54 ± 1.33	0.16	0.09	0.22	4.677	*P* < 0.001*
Did you avoid activities or situations because they reminded you of a stressful HMCAS work experience?	2.18 ± 1.25	2.35 ± 1.32	0.16	0.09	0.23	4.509	*P* < 0.001*
Did you have trouble remembering important parts of a stressful HMCAS work experience?	2.15 ± 1.17	2.31 ± 1.29	0.17	0.11	0.22	5.820	*P* < 0.001*
Did you experience a loss of interest in things that you used to enjoy?	2.59 ± 1.36	2.93 ± 1.4	0.34	0.24	0.44	6.858	*P* < 0.001*
Did you feel distant or cut off from other people?	2.45 ± 1.34	3.04 ± 1.47	0.59	0.48	0.71	10.252	*P* < 0.001*
Did you feel emotionally numb or unable to have loving feelings for those close to you?	2.13 ± 1.32	2.54 ± 1.49	0.41	0.31	0.51	8.265	*P* < 0.001*
Did you feel as if your future would somehow be cut short?	2.34 ± 1.33	2.79 ± 1.43	0.46	0.36	0.56	8.898	*P* < 0.001*
Did you have trouble falling or staying asleep?	2.57 ± 1.38	2.92 ± 1.44	0.35	0.25	0.44	7.234	*P* < 0.001*
Did you feel irritable or have angry outbursts?	2.37 ± 1.29	2.79 ± 1.37	0.42	0.32	0.51	8.549	*P* < 0.001*
Did you have difficulty concentrating?	2.08 ± 1.21	2.47 ± 1.36	0.38	0.29	0.47	8.584	*P* < 0.001*
Have you been “super alert” or watchful on guard?	2.77 ± 1.29	3.03 ± 1.32	0.26	0.18	0.35	6.430	*P* < 0.001*
Did you feel jumpy or easily startled?	2.23 ± 1.16	2.43 ± 1.22	0.20	0.13	0.28	5.386	*P* < 0.001*
**Total score out of 85**	**37.65 ± 16.03**	**42. 52 ± 17.28**	**4.87**	**3.94**	**5.80**	**10.289**	*P* < 0.001*

*Significant at *P* ≤ 0.05; SD: *s*tandard deviation.

**Table 5.** Participants’ overall level of stress based on the impact of stressful work experiences.

PTSD symptom severity (score range)	Before COVID-19 (*n* = 272)	During COVID-19 (*n* = 272)	McNemar-Bowker test	*P*-value
	n (%)	n (%)		
Little/no symptoms (17–29)	107 (39.3%)	77 (28.3%)	48.725	*P* < 0.001*
Moderate symptoms (30–44)	75 (27.6%)	75 (27.6%)	
Severe symptoms (45–85)	90 (33.1%)	120 (44.1%)	
**Total**	**272 (100%)**	**272 (100%)**	

Note: PTSD symptom severity categories and score ranges are based on the PTSD Checklist for DSM-IV (PCL-M).^25^ *Significant difference at *P* ≤ 0.05.
